# A82 DIETARY PROTEIN COMPOSITION ALTERS INTESTINAL INFLAMMATION AND MTOR ACTIVATION

**DOI:** 10.1093/jcag/gwae059.082

**Published:** 2025-02-10

**Authors:** L E Rondeau, P Muppidi, B Da Luz, K Kan, R Dang, X Wang, A Caminero

**Affiliations:** Medicine, McMaster University, Oakville, ON, Canada; Medicine, McMaster University, Oakville, ON, Canada; Medicine, McMaster University, Oakville, ON, Canada; McMaster University, Hamilton, ON, Canada; McMaster University, Hamilton, ON, Canada; Medicine, McMaster University, Oakville, ON, Canada; Medicine, McMaster University, Oakville, ON, Canada

## Abstract

**Background:**

Environmental factors, notably changes in diet and microbiota composition, have been identified as key contributors to the development of inflammatory bowel diseases (IBD). The prevalence of IBD is on the rise, particularly in industrialized nations with populations consuming western-style diets rich in fat and protein. While total and animal protein intake have been associated with IBD, their specific impact on intestinal inflammation remains poorly understood. Branch-chain amino acids found in different protein source possess bioactive properties and can interact with the mechanistic target of rapamycin (mTOR), a cellular growth regulator that controls autophagy and inflammation. Given the observed mTOR hyperactivation in IBD patients, coupled with its correlation with exacerbated colitis severity in murine models, a comprehensive exploration into the relationship between dietary protein intake, mTOR activation, autophagy, and intestinal inflammation is warranted.

**Aims:**

To study the influence of high protein and animal protein diets on mTOR activation, autophagy, and intestinal inflammation in mouse models of colitis.

**Methods:**

Specific pathogen free C57BL/6 mice were fed a high protein diet (HPD; 40% casein), animal meat protein diet (APD; 14% mixed protein), or control diet (CD; 14% casein), for three weeks prior to tissue collection (N=6 per diet) or experimental colitis induction (N=8 per diet). Experimental colitis was induced using dextran sulfate sodium in drinking water (DSS; 2.5% w/v) or colonic administration of 2,4,6-trinitrobenzene sulfonic acid (TNBS; 2%). DSS was provided *ad libitum* for five days, followed by two days of water recovery before tissue collection. Rapamycin (10 mg/kg/day) was provided intraperitoneally to a subset of mice as an mTOR inhibitor. mTOR activation and autophagy markers were assessed by western blot (WB) of mTOR substrates and autophagy proteins in colon tissue extracts pre- and post-colitis. Apoptotic cells in colon cross sections were quantified by TUNEL. Susceptibility to colitis was assessed by histologic analysis of distal colon sections, weight loss, and clinical scores. Inflammatory gene transcripts were quantified in colon tissue by Nanostring.

**Results:**

DSS and TNBS exposure in mice fed the HPD and APD led to greater weight loss, tissue damage, inflammatory gene signaling, diarrhea, and stool blood. HPD and APD increased mTOR activation (phosphorylation of mTOR and S6 ribosomal kinase) compared to CD. Autophagy proteins were downregulated, and apoptotic cells were increased in intestinal tissue of HPD- and APD-fed mice, before colitis. Inhibition of mTOR with rapamycin restored autophagy and reduced colitis severity.

**Conclusions:**

Protein quantity, source, and BCAA content optimization is crucial for determining inflammation, mTOR activation, and autophagy in mouse models of colitis.

**Funding Agencies:**

CAG, CCC, CIHR

